# Archaea and their interactions with bacteria in a karst ecosystem

**DOI:** 10.3389/fmicb.2023.1068595

**Published:** 2023-02-06

**Authors:** Xiaoyu Cheng, Xing Xiang, Yuan Yun, Weiqi Wang, Hongmei Wang, Paul L. E. Bodelier

**Affiliations:** ^1^State Key Laboratory of Biogeology and Environmental Geology, China University of Geosciences, Wuhan, China; ^2^School of Environmental Studies, China University of Geosciences, Wuhan, China; ^3^Department of Microbial Ecology, Netherlands Institute of Ecology (NIOO-KNAW), Wageningen, Netherlands; ^4^College of Life Science, Shangrao Normal University, Shangrao, China; ^5^College of Life Sciences, Nankai University, Tianjin, China

**Keywords:** karst ecosystem, karst soil, subterranean cave, archaeal community, interaction between archaea and bacteria, niche differentiation

## Abstract

Karst ecosystems are widely distributed around the world, accounting for 15–20% of the global land area. However, knowledge on microbial ecology of these systems does not match with their global importance. To close this knowledge gap, we sampled three niches including weathered rock, sediment, and drip water inside the Heshang Cave and three types of soils overlying the cave (forest soil, farmland soil, and pristine karst soil). All these samples were subjected to high-throughput sequencing of V4-V5 region of 16S rRNA gene and analyzed with multivariate statistical analysis. Overall, archaeal communities were dominated by *Thaumarchaeota*, whereas *Actinobacteria* dominated bacterial communities. *Thermoplasmata*, *Nitrosopumilaceae*, *Aenigmarchaeales*, *Crossiella*, *Acidothermus*, and *Solirubrobacter* were the important predictor groups inside the Heshang Cave, which were correlated to NH_4_^+^ availability. In contrast, *Candidatus* Nitrososphaera, *Candidatus* Nitrocosmicus, *Thaumarchaeota* Group 1.1c, and *Pseudonocardiaceae* were the predictors outside the cave, whose distribution was correlated with pH, Ca^2+^, and NO_2_^−^. Tighter network structures were found in archaeal communities than those of bacteria, whereas the topological properties of bacterial networks were more similar to those of total prokaryotic networks. Both chemolithoautotrophic archaea (*Candidatus* Methanoperedens and *Nitrosopumilaceae*) and bacteria (subgroup 7 of *Acidobacteria* and *Rokubacteriales*) were the dominant keystone taxa within the co-occurrence networks, potentially playing fundamental roles in obtaining energy under oligotrophic conditions and thus maintaining the stability of the cave ecosystem. To be noted, all the keystone taxa of karst ecosystems were related to nitrogen cycling, which needs further investigation, particularly the role of archaea. The predicted ecological functions in karst soils mainly related to carbohydrate metabolism, biotin metabolism, and synthesis of fatty acid. Our results offer new insights into archaeal ecology, their potential functions, and archaeal interactions with bacteria, which enhance our understanding about the microbial dark matter in the subsurface karst ecosystems.

## 1. Introduction

Karst ecosystems largely developed in the exposed area of soluble rocks including limestone, dolomite and gypsum, are widely distributed around the world, accounting for about 15–20% of the global land area (De Waele et al., 2009; Chalikakis et al., 2011; Di Maggio et al., 2012; Kaufmann, 2014). The overlying soil of the karst zone contains abundant organic carbon and high microbial activity in the topsoil layer (0–30 cm) (Ahmed et al., 2012). Next to this, karst forest soils show higher decomposition, higher respiration rate, but are more carbon-limited compared to non-karst forest soils (Chen et al., 2018). Except organic matter, karst overlying soils also show high nitrogen concentration even up to nitrogen saturation especially in southwestern China (Wen et al., 2016; Chen et al., 2018). Water is the bridge linking the surface karst soils and subterranean karst caves, which transports natural organic matter and trace metals into the underground ecosystem (De Waele et al., 2009; Hartland et al., 2012). δ^18^O of cave drip water is employed to track the processes of speleothem deposits (Bradley et al., 2010), which reflects meteoric precipitation in cool climate (mean annual temperature < 10°C) (Baker et al., 2019). Inside karst caves, the permanent darkness, saturated humidity, and potential evaporation largely differ from those in surface habitats in terms of light, energy, nutrients, weathering degree, and abiotic stability, which provide a unique oligotrophic habitat for biotic communities (Gabriel and Northup, 2013).

Studies have clearly demonstrated the evolutionary differences between macro-biota in subsurface and surface ecosystems. Underground macro-creatures evolve unique characteristics compared with surface creatures, which are solely found in a single cave or adjacent caves due to geographical isolation (Ribera et al., 2019). However, most microorganisms in caves can also be found in other ecosystems, but with different relative abundances. Quantitatively, prokaryotes inside the caves are 2–3 orders of magnitude lower than those in overlying karst soils. 16S rRNA gene copies ranged from 10^10^ to 10^12^ copies·g^−1^ soil in karst soils with different land use (croplands and wetland) (Hu et al., 2018; Liao et al., 2018; Wang X. et al., 2021). In contrast, the absolute abundance of 16S rRNA gene inside caves varies from 10^5^–10^9^ copies·g^−1^ weathered rock to 10^8^–10^9^ copies·g^−1^ sediment (Alonso et al., 2018; Zhao et al., 2018).

The knowledge on bacterial communities in karst ecosystems has increased recently about their involvement in elemental cycles and functional diversity. *Actinobacteria*, *Proteobacteria*, and *Acidobacteria* dominate in karst soils with different land-use (Liao et al., 2018; Cheng et al., 2021). For instance, the carbon-fixing bacterial communities of *Thiomonas*, *Bradyrhizobium*, *Ferriphaselus* and *Sulfuricaulis* are, respectively, dominant in native wetland soil, degraded wetland soil, and reclaimed farmland wetland soil of karst zone, which closely correlate to edaphic parameters such as organic carbon, dissolved organic carbon, microbial biomass carbon, and labile organic carbon (Wang X. et al., 2021). Inside karst caves, microbial diversity and elemental cycles microbes involved vary with the cave lithology. Sulfur metabolism is mostly reported in plutonic caves rich in hydrogen sulfide, where bacteria can utilize sulfur to provide metabolic energy for other cave biota (Engel et al., 2010). A large proportion of sulfate-reducing bacteria (SRB), such as *Desulfovibrio* and *Desulfomicrobium* has also been observed in Altamira Cave, suggesting potential function of sulfur reduction in supergene caves (Portillo and Gonzalez, 2009). *Acidithiobacillus*, dominated a biofilm in acid Frasassi Cave, is capable of carbon fixation, sulfur oxidation, and nitrogen assimilation, indicating the coupling of carbon and nitrogen cycling (Jones et al., 2012). Cave bacteria living on weathered rock and speleothem surfaces can fix carbon *via* Calvin-Benson-Bassham cycle, whereas those in sediments prefer the 3-hydroxypropionate/4-hydroxybutyrate (HP/HB) pathway (Ortiz et al., 2014; Yun, 2018). Furthermore, it has been demonstrated that carbon fixation by *Bacillus*, *Actinomycetes*, and *Burkholderia* increase with elevated CO_2_ concentration (Ortiz et al., 2014; Yun, 2018). Other than carbon fixation, microbes can also mediate methane cycling in caves. Recently, it has been reported that high-affinity methane-oxidizing bacteria, especially those belonging to upland soil cluster *γ* (USC*γ*), are widely distributed in karst caves from southwest and central China. The *pmoA* gene abundance of USC*γ* in weathered rocks is higher than that in sediments (Zhao et al., 2018; Cheng et al., 2021, 2022). USC*γ* is a keystone taxon in co-occurrence networks of both methane functional groups and the total bacterial communities (Cheng et al., 2021). *Nitrosococcaceae* wb1-P19, *Rokubacteriales*, *Gaiellales*, and *Nitrospira* are identified as the keystone members in bacterial networks in the karst caves, which are closely linked to elemental cycles (Zhu et al., 2019; Ma et al., 2021). Fungi dominated by *Ascomycota* played a fundamental role in maintaining the microbial ecosystem on weathered rocks inside the karst cave as indicated by the cross-domain fungi-bacteria network analysis (Wang Y. et al., 2022).

In comparison to bacteria, archaea are far less studied in karst ecosystems. Recently, abundant archaeal taxa have been detected in iron-manganese cave deposits, which harbor functional genes involved in nitrogen cycling such as nitrification, dissimilatory nitrate reduction, assimilatory nitrate reduction, and denitrification (Kimble et al., 2018). The absolute abundance of ammonia-oxidizing archaea (AOA) exceeds that of ammonia-oxidizing bacteria (AOB) up to 2 orders of magnitude in cave sediments (Zhao et al., 2016). *Thaumarchaeota* at least contribute >40% to ammonia oxidation, suggesting archaea play a vital role in cave nitrogen cycle (Zhao et al., 2016). Nitrogen availability is inextricably linked to the presence of archaea and low nitrogen content increases the abundance of *Thaumarchaeota* (> 15%) (Northup et al., 2003; Hershey and Barton, 2018). Nevertheless, we still know little about archaeal distribution in different niches in karst ecosystems and how they correlate with environmental variables.

Besides microbial functional groups mediating elemental cycling, microbial interactions also play fundamental roles in maintaining the functional stability of an ecosystem. It has been demonstrated that interactions between microbial groups rather than microbial relative abundance and alpha diversity are crucial to function in the complex engineering ecosystem (Zhao et al., 2022). The well-known interaction between archaea and bacteria is the anaerobic methanotrophic archaea (ANME) with SRB. They cooperated with each other to utilize methane as the main energy source under anaerobic condition in methane seeps (Xin et al., 2022; Yu et al., 2022). Moreover, interactions between bacteria and archaea can help them to adapt the environments with heavy metal contaminations (Li et al., 2017). Other than cooperation between archaea and bacteria, competition also happens. For example, under low NH_4_^+^ and low dissolved O_2_ conditions, AOA would compete out their counterpart AOB, and collaborate better with anammox bacteria on treating low strength nitrogen sewage (Pan et al., 2016). However, up to date, there are still knowledge gaps about archaeal functions and how archaea interact with their bacterial counterpart to sustain the karstic ecosystem.

To fill the knowledge gaps in archaeal ecology and interactions between archaea and bacteria in karst ecosystems, we sampled the forest soil, farmland soil, and pristine karst soil overlying the investigated the Heshang Cave, weathered rock, sediment, and drip water inside the Heshang Cave, central China (Yun et al., 2016). Subsequently, all these samples were sequenced for 16S rRNA gene of V4-V5 region *via* high-throughput sequencing. The purposes of this study were to (i) reveal structural differences of archaeal and bacterial communities in karst soils and inside subsurface caves, (ii) explore relationships of archaea and bacteria with environmental factors, and (iii) understand the interaction between archaea and bacteria in karst soils and inside caves.

## 2. Materials and methods

### 2.1. Sampling description and physicochemical analysis

The 250-meter long Heshang Cave (29°40′–30°48′ N and 108°30′–111°20′ E) is a dolomite karst cave formed in Cambrian, located in Changyang County, Hubei Province, central China. The Heshang Cave is about 30 m above the water surface of the Qingjiang River with a sole entrance (Supplementary Figure 1). The average annual temperature and average precipitation are about 16.5°C and 1,118 mm, respectively, in this region (Yun et al., 2016). The highest temperature in this region is observed in July (27.5°C) and the lowest temperature is in January (4.8°C). *Pinus massoniana* and *Cinnamomum bodinieri* are dominant vegetation on the top of the Heshang Cave, whereas part of the land is reclaimed to grow potato, clover, and economical crop oilseed rape (Cheng et al., 2021). Soils overlying the Heshang Cave are divided into forest soil (FS), farmland soil (FLS), and pristine soil (PS) on the basis of land-use patterns. Surface soils were collected at a depth of <10 cm by the standard five-point sampling technique. Samples at one site were collected from the 4 vertexes of a quadrangle with an area around 1 m^2^ and the central point. These samples were pooled together and homogenized. Subsequently, three subsamples were collected from the homogenized sample and served as the biological triplicate. Samples inside the cave include drip water (DW), sediments (S), and weathered rocks (W). The weathered rocks and sediments of photic zone (near the entrance), twilight zone, and dark zone were sampled separately inside cave. Surface sediment samples with a depth of 1 cm were collected with five-point sampling technique and homogenized before loading into the 50 mL sterile falcon tubes. The drip water was collected with sterile 10 L buckets and funnels, and immediately filtered with 2 μm membrane after collection. Filters with cells were preserved in sterile falcon tubes on dry ice. In total, 10 samples outside the cave (containing 4 FS, 3 FLS, and 3 PS samples), and 21 samples (containing 3 DW, 9 S and 9 W samples) inside cave were collected in the Heshang Cave karstic system. All samples were transported to the geomicrobiology lab at China University of Geosciences (Wuhan) on dry ice within 24 h and stored at −80°C upon arrival until further use. Environmental factors, such as pH, TOC (total organic carbon), Ca^2+^, Mg^2+^, K^+^, Na^+^, NH_4_^+^, Cl^−^, NO_2_^−^, NO_3_^−^, and SO_4_^2−^ concentrations were measured as reported in our previous work (Yun et al., 2016).

### 2.2. DNA extraction and sequencing processes

Total DNA of 0.5 g solid samples was extracted with the Power Soil® DNA Isolation Kit (MOBIO, United States), and total DNA from drip water was extracted from membrane filters with the Power Water® DNA Isolation Kit (MOBIO, United States). DNA quality and purification steps were executed as described previously (Ma et al., 2021). The prokaryotic universal primers 707f (3’-ATTAGATACCCSBGTAGTCC-5′) and 1059r (3’-GCCATGCACCWCCTCT-5′) were used to amplify V4-V5 region of 16S rRNA (Kim et al., 2018). Raw sequences were obtained from an Illumina MiSeq platform in two separate runs at Shanghai Personal Biotechnology, Co., Ltd., (Shanghai, China) and deposited in the National Omics Data Encyclopedia (NODE)^1^ with the project numbers OER254939.

Raw sequences were processed with the bcl2fastq software (version 1.8.4, Illumina) to cut primers and barcodes. Subsequently, the quality and analysis of processed sequences were controlled with QIIME2 (Quantitative Insight Into Microbial Ecology, version 2019.7) software (Bolyen et al., 2019). The high-quality sequences were clustered at 97% sequence similarity to generate representative OTU (Operational Taxonomic Unit) sequences with the VSEARCH plugin, and the feature taxonomy of the 16S rRNA gene was assigned against modified SILVA database (version 132) to improve the efficiency of archaeal annotation. The sequence numbers of all samples were resampled to 60,000 reads to avoid the influence of sequencing depth on microbial diversity.

### 2.3. Statistical analysis

Shannon index for each sample, beta diversity of archaea and bacteria, permutational multivariate analysis of ANOVA (ADONIS), analysis of similarities (ANOSIM), and multi response permutation procedure (MRPP), mantel test, and principal coordinate analysis (PCoA) were calculated with the vegan^2^ package in R software (version 4.0.3). The ADONIS, ANOSIM, and MRPP analysis of microbial community were performed based on Bray-Curtis distance, and the normalized *p* values of these analysis were adjusted by Benjamini and Hochberg method. Mantel test was performed to evaluate the correlation between environmental matrix and microbial matrix *via* Spearman’s rho analysis under 9,999 permutations. To further assess the spatial patterns of beta diversity, total dissimilarity of Sørensen indices (β_sor_), their turnover component (β_sim_), and their nestedness component (β_sne_) were computed *via* betapart package (Baselga and Orme, 2012). Random forest machine learning was conducted with randomForest^3^ and A3^4^ package to rank the impacts of microbial genus in cave ecosystem. To further understand functional inference and ecological trait assignment of microbial community in cave ecosystem (Djemiel et al., 2022), we, respectively, performed representative sequences with Tax4Fun2 package (Wemheuer et al., 2020) and Functional Annotation of Prokaryotic Taxa database (FAPROTAX, v1.2.3).^5^ The boxplot, pie chart, histogram, heatmap, scatter diagram, density map, and PCoA plot were visualized with ggpubr,^6^ pheatmap, and ggplot2^8^ package. The analysis of variance (ANOVA), student’s *t*-test (*t*-test), and Kruskal-Wallis *H* test were calculated with SPSS statistics (version 26.0).

The archaeal OTUs that had relative abundance above 0.01% and more than 20% occurrence in all samples and bacterial OTUs that had relative abundance above 0.01% and more than 50% occurrence were selected for co-occurrence networks analysis to reduce the network complexity with Hmisc^9^ and igraph^10^ packages. The correlations among archaeal OTUs, bacterial OTUs, and archaeal and bacterial OTUs were calculated with correlation coefficient *ρ* of ≥0.7 and *p* value of <0.01 (Benjamini and Hochberg method adjusted) based on Spearman’s *p* correlation. Networks were visualized with the layout of Fruchterman-Reingold in Gephi (version 0.9.2) software. Nodes with high betweenness centrality values indicated that a node was capable of sustaining the nodes of community and had high connectivity with other nodes, which were considered keystone taxa in the network (Martín González et al., 2010; Vick-Majors et al., 2014; Jiao et al., 2016).

## 3. Results

### 3.1. Archaeal and bacterial community composition in the cave ecosystem

Totally, a number of 1,860,000 high-quality sequences were recovered after quality control, which were converted into 43,488 OTUs containing 1,539 archaeal OTUs, 41,665 bacterial OTUs, and 283 unassigned OTUs based on 97% similarity.

**Figure 1:**
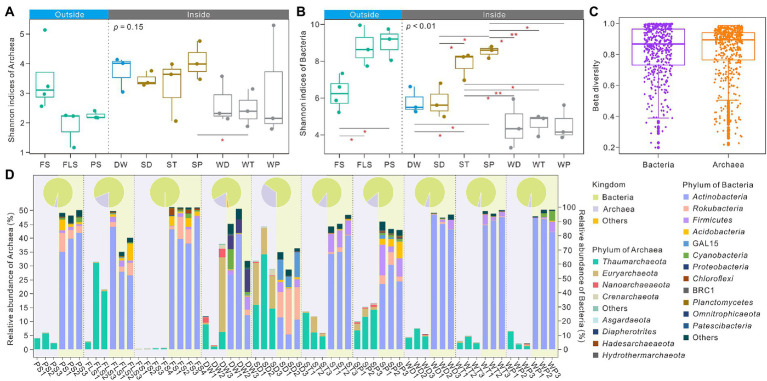
Alpha, beta diversity distribution and community composition of archaea and bacteria in cave ecosystem of the Heshang Cave, central China. Panel **(A)** and **(B)**, respectively, showed Shannon indices of archaea and bacteria in different niches inside or outside the Heshang Cave. Panel **(C)** indicated the result of beta diversity between archaea and bacteria. Panel **(D)** showed the relative abundance of archaeal phylum and bacterial phylum from different niches. Pie charts showed the relative abundance of total bacteria and archaea in an individual niche and histogram shows the relative abundances of bacterial or archaeal phyla in each sample. The relative abundance of archaea refers to the left axis scale, whereas bacterial abundance refers to the right axis scale. Histograms for archaea are located in the area highlighted in light purple, whereas those of bacteria in light green. FS, forest soil; PS, pristine soil; FLS, farmland soil; WD, weathered rocks in the dark zone; WT, weathered rocks in the twilight zone; WP, weathered rocks in the photic zone; SD, sediments in the dark zone; ST, sediments in the twilight zone; SP, sediments in the photic zone; DW, drip waters.

Significant differences of bacterial Shannon indices between FS and FLS, and between FS and PS were observed in the soils overlying the Heshang Cave, whereas archaeal Shannon indices were significantly different only between SP (sediments in the photic zone) and WT (weathered rocks in the twilight zone) inside the cave (Figures 1A,B). Shannon index showed an opposite pattern between archaea and bacteria. For instance, archaeal Shannon index was highest (3.48 ± 0.99) in FS, whereas bacterial Shannon values was lowest (6.26 ± 0.99) in overlying soils (Figures 1A,B). This phenomenon was also found inside the Heshang Cave. DW showed higher archaeal Shannon index (3.73 ± 0.49) and lower bacterial Shannon value (5.80 ± 0.59) compared with other cave niches (Figures 1A,B). Shannon indices of sediment samples were generally higher than those in weathered rock, especially in the photic zone (Figures 1A,B). Notably, no significant difference between archaeal and bacterial beta diversity was observed, whereas archaeal beta diversity was higher than that of bacteria (Figure 1C). Both archaeal and bacterial community were significantly dissimilar between overlying soils and samples inside the cave as verified by ADONIS, ANOSIM, and MRPP based on Bray-Curtis distances (Supplementary Table 1). The principal coordinate analysis (PCoA) showed that both bacterial and archaeal communities exhibit niche specificity (Supplementary Figure 2). The PCo1 and PCo2 axis explained 20 and 16% of the variance in the bacterial community, and 28 and 14% of the variance in the archaeal community (Supplementary Figure 2). The relative abundance of archaea in SD (sediments in the dark zone), DW, SP, and FS niches was higher than those in other niches. In contrast, bacterial relative abundance was higher than those of archaea in cave ecosystem (Figure 1D).

*Thaumarchaeota* dominated in all archaeal communities at the phylum level, especially in FS (18.21 ± 11.82%) and SD (21.69 ± 8.88%) niches. *Actinobacteria*, ranging from 17.79 ± 5.39% to 90.18 ± 2.31%, and *Rokubacteria*, ranging from 0.25 ± 0.13% to 24.22 ± 5.99%, were dominant in bacterial communities inside the cave (Figure 1D; Supplementary Table 2). Significant difference was observed in the relative abundance of phylum *Thaumarchaeota*, *Euryarchaeota*, *Nanoarchaeaeo*ta, *Rokubacteria*, *Firmicutes*, *Acidobacteria*, GAL15, *Proteobacteria*, *Chloroflexi*, BRC1, *Planctomycetes*, *Omnitrophicaeota*, and *Patescibacteria* between overlying soils and niches inside the cave (Supplementary Table 2). *Thaumarchaeota* and BRC1 were significantly different in their relative abundances in overlying soils with different land use. Inside the cave *Thaumarchaeota*, *Nanoarchaeaeota*, *Actinobacteria*, *Rokubacteria*, *Acidobacteria*, GAL15, *Proteobacteria*, *Chloroflexi*, BRC1, *Planctomycetes*, *Omnitrophicaeota*, and *Patescibacteria* also showed significant differences in their relative abundances (Supplementary Table 2).

**Figure 2:**
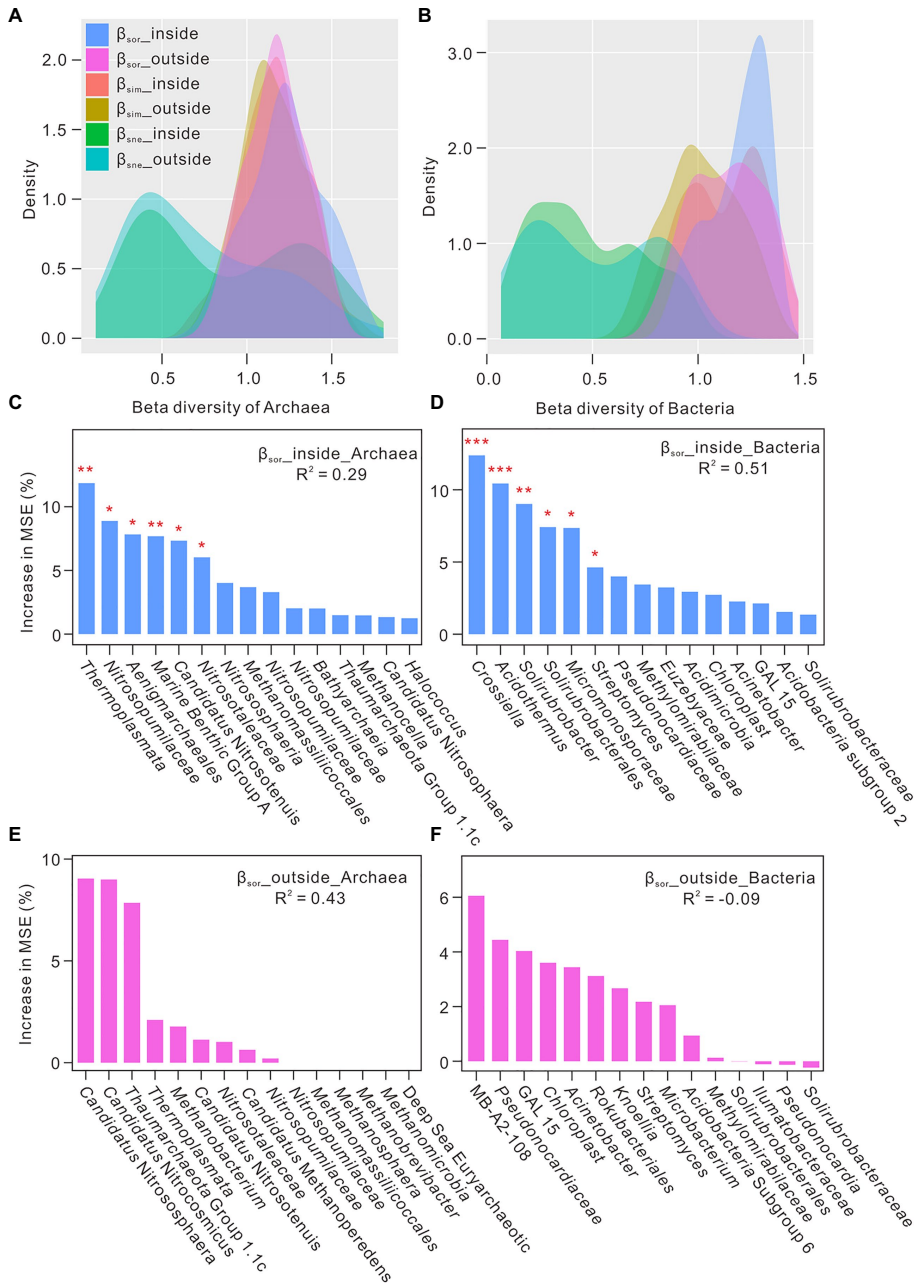
The relationship between beta diversity and microbial genus in the karst ecosystem. Panel **(A)** and **(B)** indicated the partition of β_sor_ into β_sim_ and β_sne_ inside and outside the Heshang Cave, central China. Panel **(C–F)** showed that importance ranking (percentage of increase of mean square error, MSE) of archaeal and bacterial genus under the conditions of β_sor_ values as indicated by random forest machine learning. High MSE values mean more importance of microbial genus as predictors compared with those with low MSE values. *indicated a *p* value <0.05, and **indicated a *p* value <0.01.

To infer mechanisms underlying the observed biodiversity patterns, beta diversity based on the Sørensen index were conducted to separate the turnover and nestedness components, which, respectively, indicated the processes of species replacement and species loss (or gain) in the environment (Baselga and Orme, 2012). Overall turnover components (β_sim_) were much higher than nestedness-resultant dissimilarity (β_sne_) (Figures 2A,B), which suggested the noteworthy substitution of microbial community in these niches and thus contributed to their community differences (Baselga and Orme, 2012). Random forest algorithm analysis was employed to understand the important rank of microbial genus under β_sor_ values (Figures 2C–F). Archaeal and bacterial genera with high percentage of increase of mean square error (MSE) were defined as the most important predictors of total microbial composition. *Thermoplasmata* (affiliated to phylum *Euryarchaeota*), *Nitrosopumilaceae* (*Thaumarchaeota*), *Aenigmarchaeales* (*Nanoarchaeaeota*), *Crossiella* (*Actinobacteria*), *Acidothermus* (*Actinobacteria*), and *Solirubrobacter* (*Actinobacteria*) were the important genera closely correlated to β_sor_ values inside the Heshang Cave (Figures 2C,D). *Candidatus* Nitrososphaera (*Thaumarchaeota*), *Candidatus* Nitrocosmicus (*Thaumarchaeota*), *Thaumarchaeota* Group 1.1c (*Thaumarchaeota*), MB-A2-108 (*Actinobacteria*), and *Pseudonocardiaceae* (*Actinobacteria*) were the important genera in the overlying soils (Figures 2E,F).

### 3.2. Correlations between microbial communities and environmental variables

**Figure 3:**
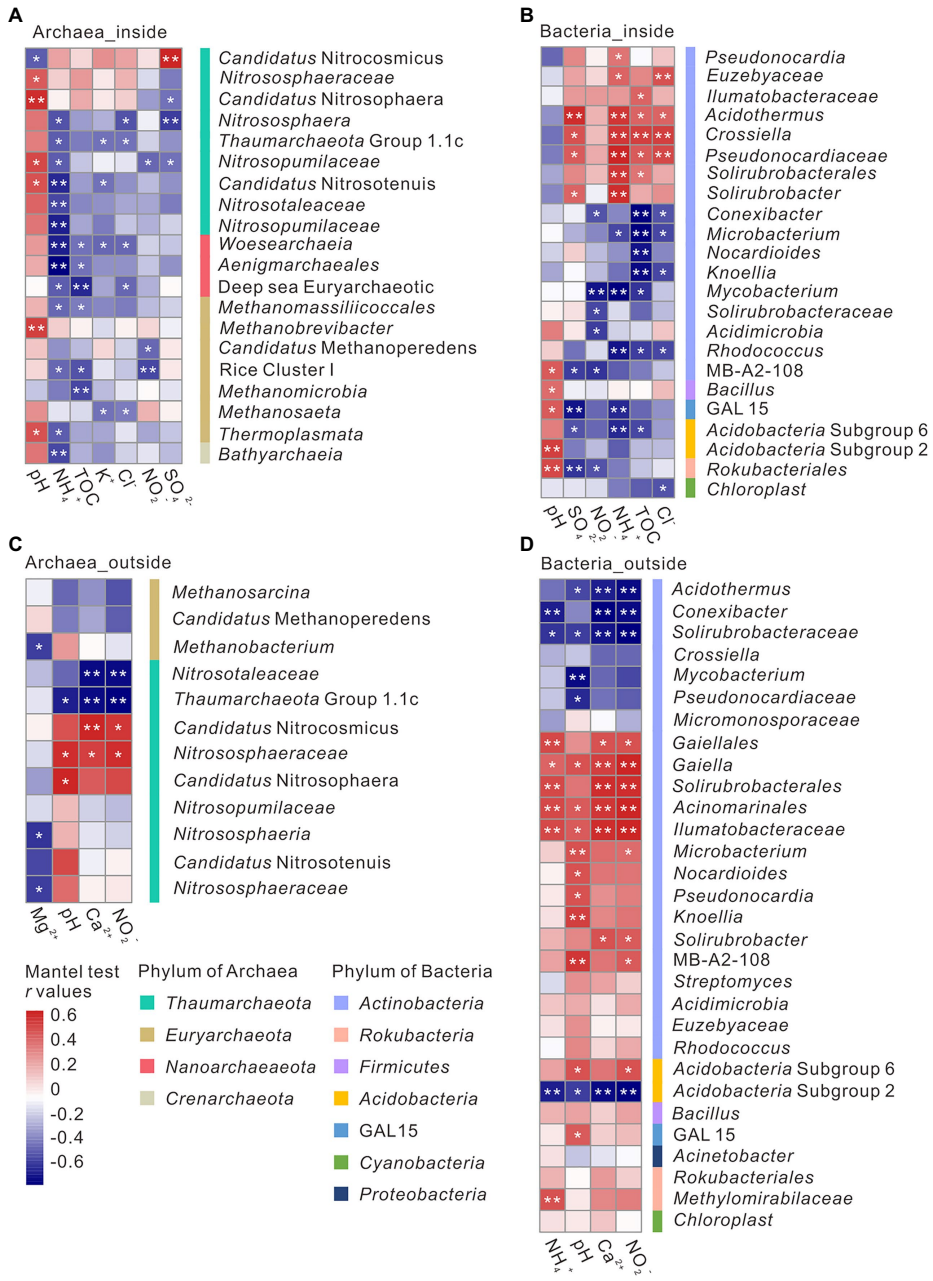
The correlations between environmental factors and microbial genus inside **(A, B)** and outside **(C, D)** the Heshang Cave under the analysis of mantel test. Positive correlations were in red and negative correlations were in blue. *indicated a *p* value <0.05, and **indicated a *p* value <0.01.

Correlations between environmental parameters and microbial community structure were analyzed *via* Mantel test. In overlying soils, both archaeal and bacterial communities were closely correlated with pH, Ca^2+^, and NO_2_^−^ concentrations, whereas bacterial communities were also correlated with NH_4_^+^ concentration (Supplementary Table 3; Figures 3C,D). The important predictor groups identified above were found to be significantly correlated with environmental variables. For instance, relative abundance of *Thaumarchaeota* Group 1.1c and *Pseudonocardiaceae* showed a negative correlation with pH, whereas the relative abundance of *Candidatus* Nitrososphaera and MB-A2-108 positively correlated with pH (Figures 3C,D). NO_2_^−^ concentration positively correlated with the relative abundance of *Candidatus* Nitrocosmicus and MB-A2-108, whereas negatively with the relative abundance of *Thaumarchaeota* Group 1.1c (Figures 3C,D). Within the cave, NH_4_^+^ concentration negatively correlated to archaeal (*Woesearchaeia*, *Thermoplasmata*, *Nitrosopumilaceae*, and *Aenigmarchaeales*) and positively correlated to bacterial communities (*Acidothermus*, *Crossiella*, *Pseudonocardiaceae*, and *Solirubrobacter*) (Figures 3A,B). Besides NH_4_^+^ concentration, the relative abundance of *Woesearchaeia* and *Aenigmarchaeales* negatively correlated with TOC content, and the relative abundance of *Nitrosopumilaceae* and *Thermoplasmata* showed a positive correlation with pH (Figure 3A). Relative abundances of *Solirubrobacter* and *Acidothermus* showed a positive correlation with SO_4_^2−^ concentration (Figure 3B).

### 3.3. Microbial interactions in the cave ecosystem

**Figure 4:**
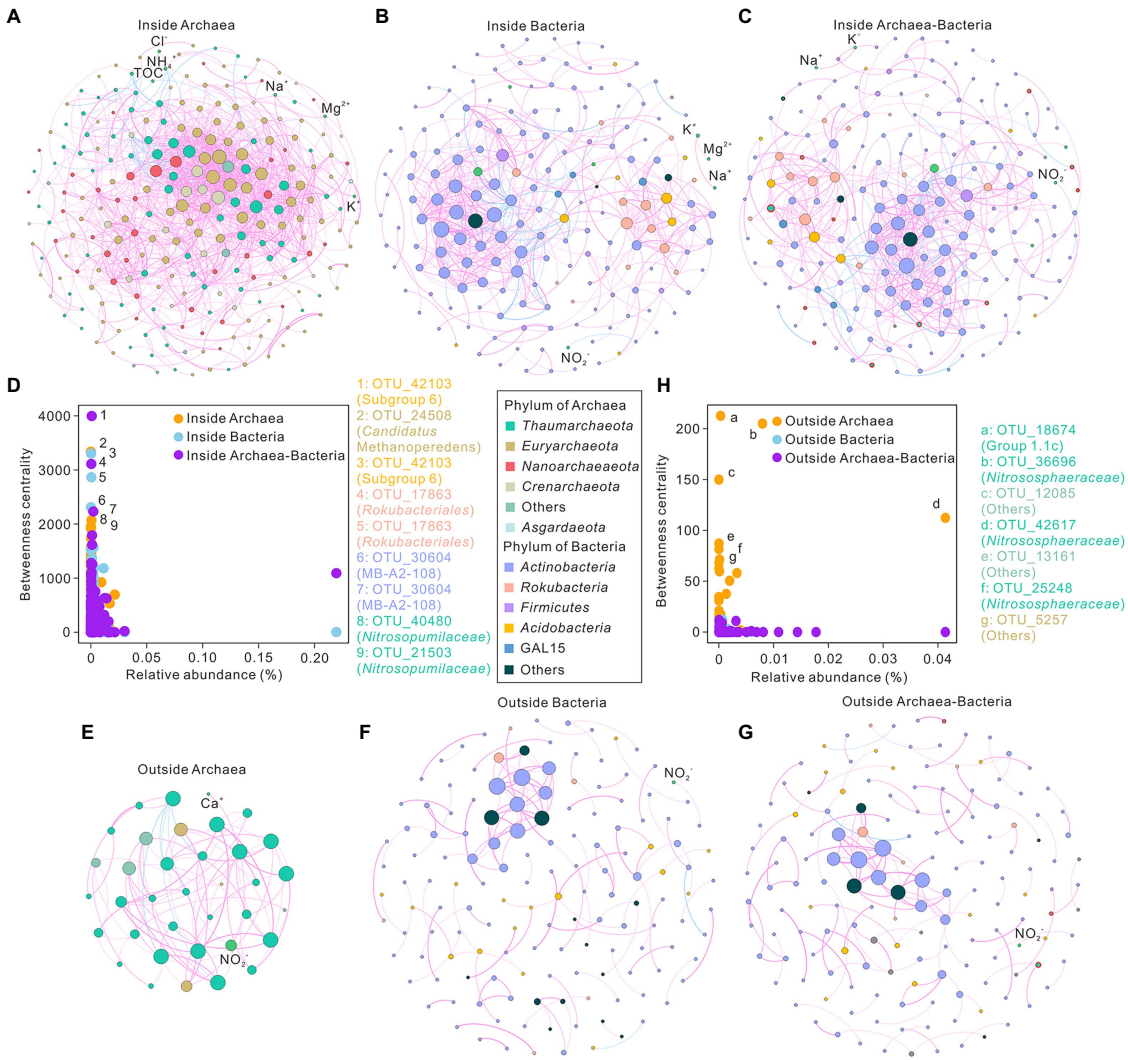
Co-occurrence networks of microbial communities in the Heshang Cave ecosystem, central China. Panel **(A–C)** indicated networks of archaeal **(A)**, bacterial **(B)**, and total prokaryotic **(C)** communities inside the Heshang Cave. Panel **(E–G)** showed networks of archaeal **(E)**, bacterial **(F)**, and total prokaryotic **(G)** communities outside the Heshang Cave. Panel **(D,H)** revealed the betweenness centrality and relative abundance of network nodes inside **(D)** and outside (H) the cave. Each node represents an OTU in the network, and the node size is proportional to the degree (connecting with other nodes). Negative links were in blue, whereas positive edges were in pink. Nodes of archaea were marked with red outer rings.

To investigate underlying interactions among archaea, bacteria, and environmental variables, co-occurrence networks were conducted in the cave ecosystem. The inside-cave networks of archaea, bacteria, and total prokaryotes had 250, 181, and 194 nodes and 1,405, 521, and 523 edges, respectively (Figures 4A–C,E–G; Supplementary Table 4). In contrast, the outside networks of archaea, bacteria, and total prokaryotes had 36, 145, and 152 nodes and 80, 128, and 132 edges, respectively (Supplementary Table 4). Positive links dominated the edges in all co-occurrence networks (Figure 4). The modularity values of all networks were higher than 0.4, showing good modular structures (Supplementary Table 4). Higher average clustering coefficient, higher density, higher average degree, lower diameter, and lower average path length were observed in archaeal topological properties compared to bacterial and total networks inside the cave. Similar patterns were also observed in topological properties of outside archaeal network with exceptions in average path length and diameter values (Supplementary Table 4). Bacterial networks and total prokaryotic networks were more similar in the topological properties of (Supplementary Table 4). Concentrations of NO_2_^−^ and Ca^2+^ were tightly correlated with nodes in the outside archaeal network (Figure 4E).

The relevance between the relative abundance of nodes and betweenness centrality revealed that keystone taxa in low relative abundance sustain the function in the co-occurrence networks (Figures 4D,H). The taxonomy of keystone taxa was significantly different between networks of total prokaryotes inside and outside the cave. Inside the cave, keystone taxa consisted of subgroup 6 (*Acidobacteria*), *Candidatus* Methanoperedens (*Euryarchaeota*), *Rokubacteriales* (*Rokubacteria*), MB-A2-108 (*Actinobacteria*), and *Nitrosopumilaceae* (*Thaumarchaeota*) (Figure 4D). In contrast, keystone taxa of the prokaryotic network outside the cave were constituted by *Solirubrobacterales*, *Ilumatobacter*, and *Gaiellales* (Figure 4H, Supplementary Table 5).

### 3.4. Microbial functional inference and ecological trait assignment in cave ecosystem

**Figure 5:**
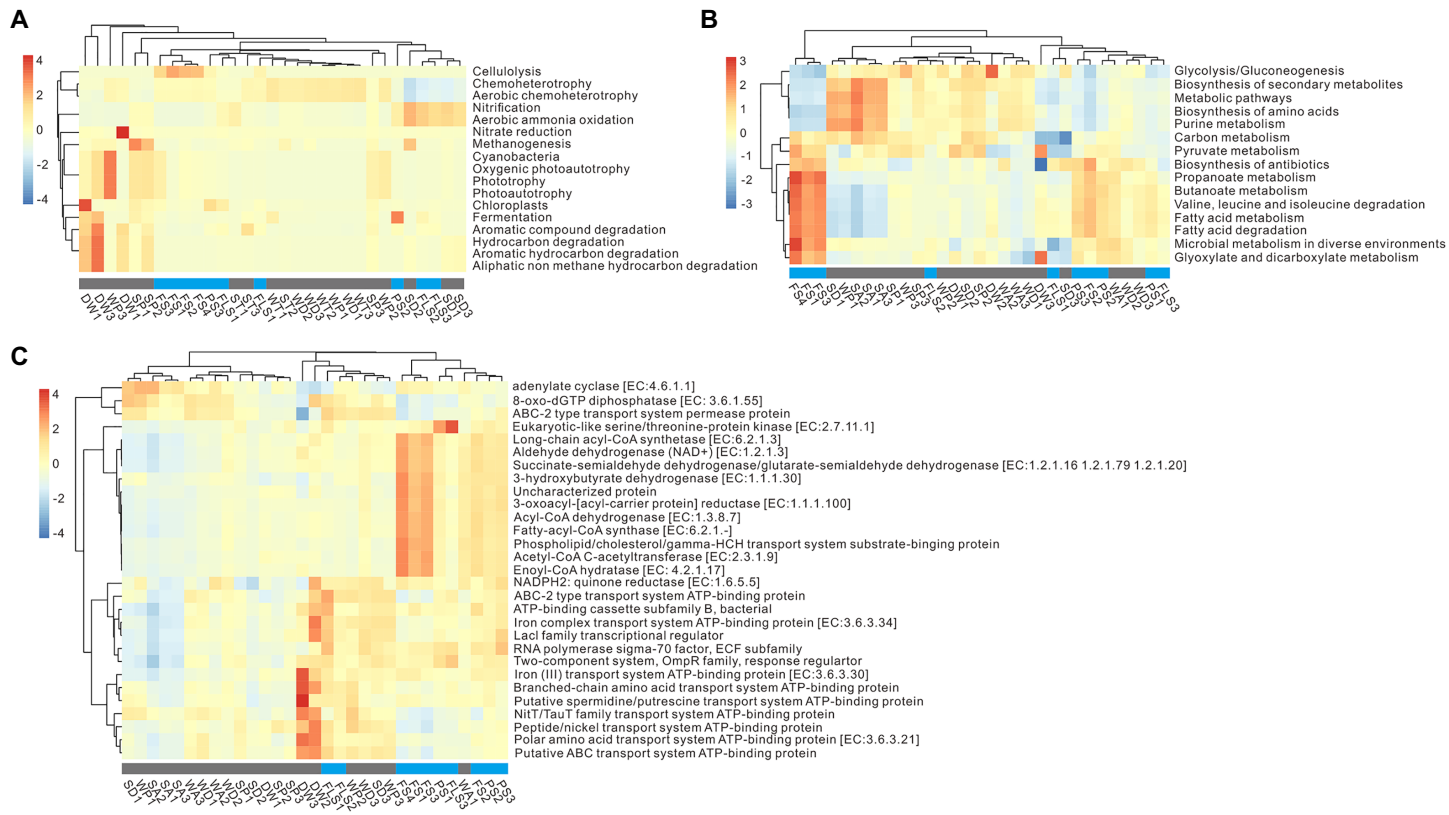
Ecological trait assignment and functional inference of microbes in the cave ecosystem. Panel **(A)** showed the top 17 results of ecological trait assignment based on the analysis of FAPROTAX. Panel **(B,C)** indicated pivotal functional inference with the Tax4Fun2 software. The relative abundance of top 15 pathways **(B)** and top 29 functional genes **(C)** in each sample. All these results were revealed with the Pearson correlation. FS, forest soil; PS, pristine soil; FLS, farmland soil; WD, weathered rocks in the dark zone; WT, weathered rocks in the twilight zone; WP, weathered rocks in the photic zone; SD, sediments in the dark zone; ST, sediments in the twilight zone; SP, sediments in the photic zone; DW, drip waters.

Microbial functional prediction consists of microbial functional inference and ecological trait assignment in general (Djemiel et al., 2022). The results of functional prediction were mainly clustered by niches (Figure 5). In the weathered rocks, chemo-heterotrophy was dominant, and WP (weathered rocks in the photic zone) particularly exhibited functional inference of cyanobacteria, oxygenic photoautotrophy, and phototrophy (Figure 5A). In sediments, SP (sediments in the photic zone) mainly showed methanogenesis, and ST (sediments in the twilight zone) and SD (sediments in the dark zone) were dominated by nitrification and aerobic ammonia oxidation, which were similar to the functions in PS and FS niches (Figure 5A). The functional inference of DW consisted of chloroplasts, fermentation, and multiple degradation of organic compounds (Figure 5A).

For the details of microbial functional inference, inside and outside niches were predominated by different metabolic pathways (Figure 5B). For specific enzymes in cave ecosystem, adenylate cyclase [EC: 4.6.1.1], 8-oxo-dGTP diphosphatase [EC: 3.6.1.55], and ABC-2 type transport system permease protein were dominant in inside the cave (Figure 5C). The proteins related to ATP binding, such as iron (III) transport system ATP-binding protein [EC: 3.6.3.30], branched-chain amino acid transport system ATP-binding protein, and putative spermidine/putrescine transport system ATP-binding protein, were dominant in DW samples (Figure 5C). Furthermore, dehydrogenase (long-chain acyl-CoA synthetase [EC: 6.2.1.3], aldehyde dehydrogenase (NAD+) [EC: 1.2.1.3], succinate-semialdehyde dehydrogenase/glutarate-semialdehyde dehydrogenase [EC: 1.2.1.16, 1.2.1.79, 1.2.1.20], and 3-hydroxybutyrate dehydrogenase [EC: 1.1.1.30]), reductase (3-oxoacyl-[acyl-carrier protein] reductase [EC: 1.1.1.100]), and synthase (Fatty-acyl-CoA synthase [EC: 6.2.1.-]) enzymes were dominant in outside samples (Figure 5C).

## 4. Discussion

### 4.1. Differences in prokaryotic communities within and outside the Heshang Cave

Overall, *Thaumarchaeota* and *Crenarchaeota* were reported to be dominant in karst niches, such as weathered rocks, sediments within the cave and soils outside the cave (Ai et al., 2022), which were consistent with our results mainly dominated by *Thaumarchaeota*, with a relative abundance ranging from 0.23 ± 0.14% to 21.69 ± 8.88% (Figure 1D; Supplementary Table 2). *Thaumarchaeota* might associate with the acidic environment due to their dominance in the acidic cave with a pH value ranging from 4.97 to 6.88 (Barton et al., 2014). However, wide occurrence of *Thaumarchaeota* was also observed in the biofilm on weathered rocks and stalactites in southwestern karst caves (Anda et al., 2017; Dong et al., 2020). High abundance of *Thaumarchaeota* was observed in karst farmland soils outside the cave (Figure 1D), and other farmland soils with low salinity and with heavy metal pollutions (Zheng et al., 2017; Chang et al., 2021; Hu et al., 2021). The bacterial community was dominated by *Actinobacteria* (Figure 1D; Supplementary Table 2). *Actinobacteria* are widely distributed in karst ecosystems (Fan et al., 2019; Zhu et al., 2019; Buresova-Faitova et al., 2022), and the Actinobacterial communities were significantly different between human-influenced and pristine karst caves, which were, respectively, predominated by *Nocardia*, *Mycobacterium*, and *Gaiellales* (Buresova-Faitova et al., 2022).

It should be pointed out that significant differences between microbial communities inside and outside the karst cave may result from their unique habitat characteristics. *Euryarchaeota* was predominant in DW3, located in the twilight zone, and in sediment samples across the cave, especially in the dark zone (Figure 1D). *Euryarchaeota* closely correlated with the functions of sulfur reducers, sulfate reducers, and thermophilic heterotrophs, and had been found on the surface of bat guano pile (Chroňáková et al., 2009). The sediments in the dark zone of the Heshang Cave were covered with bat guano, the dominance of *Euryarchaeota* in our cave was consistent with previous studies. Simultaneously, high Shannon indices of archaea and bacteria were observed in the Heshang Cave ecosystem (Figures 1A,B) compared with the previous reports (Ai et al., 2022; Xiao et al., 2022). SD was dominant by *Rokubacteria* (24.22 ± 5.99%) and GAL15 (13.03 ± 4.29%) (Figure 1D, Supplementary Table 2). The potential anaerobic conditions in SD samples might account for the dominance of the phylum *Rokubacteria*, which might be capable of anaerobic methane oxidation coupling with nitrite reduction (Lomakina et al., 2019; Gonzalez-Pimentel et al., 2021). GAL15 was previously proposed as rare cave biosphere due to their rare occurrence in caves (Gonzalez-Pimentel et al., 2021). However, the relative high abundance of GAL15 (13.03 ± 4.29%) in sediments in the dark zone in the Heshang Cave potentially indicated their important role in cave ecosystem, which merits further investigation.

The results of beta diversity showed that microbial community at one site may substitute by different microbes from other sites (Baselga and Orme, 2012), which may diversify microbial community structures in karst cave ecosystems (Figures 2A,B). Inside the cave, archaeal groups such as *Thermoplasmata*, *Nitrosopumilaceae*, and *Aenigmarchaeales* (Figure 2C), and bacterial groups such as *Crossiella*, *Acidothermus*, and *Solirubrobacter* (Figure 2D) were the important taxa closely correlated to β_sor_ values, revealing their vital roles in these ecosystems. *Thermoplasmata*, one of acidophilic archaea, is reported to be present in the snottiest from the sulfide-rich Frasassi Cave (Macalady et al., 2007). Members of this group are considered only living in the aphotic environment, and played a role on obligate heterotrophs of fermenting the breakdown products of cellular decomposition with SO_4_^2−^ as an electron acceptor (Barton and Luiszer, 2005). Our detection of *Thermoplasmata* with high relative abundance of 10.50 ± 2.78% in the alkaline Heshang Cave, especially in sediments of dark zone indicated their wider ecological niches in natural environments than previously thought. Their ecological functions in alkaline caves merits further investigation. *Nitrosopumilaceae* were reported to live in the active and fossil parts of the Dupnisa Cave System with the relative abundance >5% (Doğruöz-Güngör, 2020). *Nitrosopumilaceae* preferred aquatic niches (DW) in the Heshang Cave, which might process ammonia-oxidation in water samples (Toumi et al., 2021). *Aenigmarchaeales,* found in ice-capped lake and wheat-maize rotation soils, has never been reported in karst ecosystem (Wan et al., 2021; Vigneron et al., 2022). Nevertheless, low abundance of *Aenigmarchaeales* was found in DW samples (0.31 ± 0.33%), especially in DW1 with high metabolic potential in summer (Yun et al., 2018). The functions of *Aenigmarchaeales* affiliated to anaerobic DPANN archaea contained not only degradation and fermentation of cellular compounds, and sulfide and polysulfide reduction, but also coded genes of pore-forming toxins, peptidoglycan degradation, and RNA scavenging (Vigneron et al., 2022). Four *Actinobacteria* taxa were closely correlated with beta diversity inside the cave (Figure 2D). Higher abundance of *Crossiella* was previously reported in weathered rocks than in sediments from the Heshang Cave, which were also identified as the key genera and keystone taxa in weathered rocks as indicated by the analysis of least discriminant analysis effect size and network (Yun et al., 2016; Ma et al., 2021). Recent study showed that syntrophic relationship existed between *Crossiella* and nitrifying bacteria during the processes of CO_2_ fixation, heterotrophic ammonification, and the moonmilk formation (Martin-Pozas et al., 2022). *Acidothermus*, detected in acidophilic and thermal environments, was found in weathered rock from a thermal karst cave, speleothems and mineral surface from an orthoquartzite cave (Mohagheghi et al., 1986; Borsodi et al., 2012; Ghezzi et al., 2021). *Solirubrobacter* was abundant in weathered rock in the twilight and dark zones, ranging from 6.98 ± 1.05% to 7.51 ± 2.28%, which preferred aerobic, neutral, and mesophilic (28–30°C) conditions (Singleton et al., 2003).

Outside the cave, substitution of microbial communities might be weak in comparison with those in caves as no microbial groups significantly contributed to β_sor_ (Figures 2E,F). *Candidatus* Nitrososphaera, *Candidatus* Nitrocosmicus, *Thaumarchaeota* Group 1.1c, MB-A2-108, and *Pseudonocardiaceae* were the important taxa (Figures 2E,F). *Candidatus* Nitrososphaera and *Candidatus* Nitrocosmicus are ammonia-oxidizing archaea (Wu et al., 2021), and *Candidatus* Nitrososphaera have been reported to be dominant in karst soils (Wang et al., 2019). The relative higher abundance of *Candidatus* Nitrocosmicus (1.26 ± 1.40%) in farmland soil than that in FS and PS samples may result from the N-fertilization (Tanunchai et al., 2021). High relative abundance of *Thaumarchaeota* Group 1.1c (0.08%) was observed in FS samples with a pH ranging from 4.05–5.69 (Yun et al., 2016), which coincided with their dominance in acidic soils (Tripathi et al., 2013). MB-A2-108, affiliated to phylum *Actinobacteria*, was more commonly found in farmland soils compared with other karst niches, which also lived in stony meteorites (Tait et al., 2017).

### 4.2. Different response of prokaryotic communities to environmental factors in the karst ecosystem

Microbial communities inside and outside the cave response differently to environmental factors (Supplementary Table 3). Inside the cave, pH and NH_4_^+^ significantly impacted on archaeal relative abundance, whereas multiple edaphic variables such as NH_4_^+^, TOC, SO_4_^2−^, pH, and NO_2_^−^ significantly correlated with the relative abundances of bacterial groups. pH positively impacted on the relative abundance of *Nitrosopumilaceae* and *Thermoplasmata*, which dominated in DW and sediment samples with a pH of 7.76–8.35 (Figure 3A). The relationship between *Nitrosopumilaceae* and pH was consistent with those observed in farmland soils and saline soils (Wan et al., 2021; Wang B. et al., 2022). The relative abundance of *Thermoplasmata* was reported negatively correlated with pH in acidic tropic soil, acidic springs, solfataras, and volcanic soils (Tripathi et al., 2013), whereas an opposite correlation was observed in our alkaline cave. The detection of *Thermoplasmata* in anthropogenic reservoir of salino-alkaline lime (Kalwasinska et al., 2019) supported their occurrence in alkaline environments. Besides pH, TOC and NH_4_^+^ concentration also significantly impact the relative abundances of archaeal groups inside the cave. The relative abundance of *Woesearchaeia* and *Aenigmarchaeales* negatively correlated to TOC (Figure 3A), which dominated in DW samples with a relative abundance of 1.31 ± 1.09% and 0.31 ± 0.33%, respectively (Supplementary Table 2). These anaerobes might be brought into the cave by drip water from the anoxic overlying rocks, which performed nutrient complementation with other microbes due to their prominent metabolic shortages (Castelle et al., 2015; Liu et al., 2018; Vigneron et al., 2022; Xiang et al., 2022). NH_4_^+^ concentration significantly impacted on the relative abundances of many archaeal and bacterial groups in the Heshang Cave (Figures 3A,B), such as the important taxa of *Thermoplasmata*, *Nitrosopumilaceae*, *Aenigmarchaeales*, *Crossiella*, *Acidothermus*, and *Solirubrobacter*, indicating the significance of NH_4_^+^ in cave ecosystems. Pivotal chemolithotrophy of sulfur- and ammonium-microbes, dominated by *Nitrospira* and *Candidatus* ‘Nitrotoga’, was reported in Movile Cave (Chen et al., 2009). Except hypogene caves, active nitrification has also be detected from cave sediments dominated by ammonia-oxidizing archaea in supergene karst cave (Zhao et al., 2016).

Microbial communities outside the cave closely correlated to pH and NO_2_^−^ concentration (Figures 3C,D). pH has been reported to shape microbial communities in soils. However, different microbial groups show different response to pH variation. The relative abundance of Group 1.1c was positively correlated to pH (Figure 3C), which has been found in acidic soils across a pH gradient from 4.5 to 7.5 (Lehtovirta et al., 2009; Tripathi et al., 2013). The relative abundance of *Pseudonocardiaceae* negatively correlated with pH, whereas MB-A2-108 positively correlated with pH (Figures 3C,D) (Seuradge et al., 2017), which was consistent with our results. *Pseudonocardiaceae* has been isolated within a pH range of 5.0–11.0 (Basilio et al., 2003), but they grow better with neutral pH (Ningsih et al., 2019). *Candidatus* Nitrososphaera, positively correlated to pH, was isolated from arable soil with a pH of 7.5 (Ningsih et al., 2019; Nan et al., 2020), and potentially capable of ammonia oxidation as indicated by their genome information (Spang et al., 2012). NO_2_^−^ concentration positively impacted on the relative abundances of MB-A2-108 and *Candidatus* Nitrocosmicus (Figures 3C,D), of which *Candidatus* Nitrocosmicus could oxidize ammonia in high concentration (Lehtovirta-Morley et al., 2016; St. Clair et al., 2020).

### 4.3. Interactions between bacteria and archaea and potential microbial functions in the karst ecosystem

Strong interactions between archaea and bacteria were observed inside the cave (Figure 4C) as indicated by the visibly direct links between microbial groups in these two domains in the co-occurrence network. For instance, archaeal OTU_42617 (affiliated to *Nitrososphaeraceae*) showed positive correlations with bacterial groups such as *Rokubacteriales*, GAL15, *Gaiella*, Acidobacterial subgroup 6, and subgroup 9 (Figure 4C). All these taxa are reported to be able to participate in nitrogen cycle (Becraft et al., 2017; de Chaves et al., 2019; Obermeier et al., 2020; Longepierre et al., 2021). *Rokubacteriale* participated in nitrogen respiration (Becraft et al., 2017) and *Gaiella* contributed to the conversion of nitrate to nitrite (Albuquerque et al., 2011; Lv et al., 2022). Moreover, GAL15, acidobacterial subgroup 6, and subgroup 9 had been reported to be sensitive to nutrient availability (Hester et al., 2017; Wang J. et al., 2021). Therefore, nitrogen cycle might serve as the vital point in the interaction of archaea and bacteria inside cave, which merits further investigation.

In the prokaryotic networks putative ammonia oxidizer *Nitrososphaeraceae* and nitrate reducer *Gaiella* were found positively correlated both inside and outside the cave (Figures 4C,G), which is also reported in the urban and agricultural ditches with nitrogen-removal (Tatariw et al., 2021). The association between *Nitrososphaeraceae* and *Solirubrobacterales* might be correlated to nutrient flow and soil function recovery in karst soils (Figure 4G) since these taxa are found in high abundance in the highly damaged soils (Wei et al., 2020; Qiu et al., 2021).

Significant different keystone taxa were identified in the inside and outside archaea-bacteria co-occurrence networks based on the top five of betweenness centrality values, which putatively played fundamental ecological functions to sustain the ecosystems. Despite that, the topological properties of bacterial networks were similar to that of the total microbial networks (Supplementary Table 4), the potential ecological functions of archaea could not be overlooked in the karst ecosystems (Supplementary Table 4).

Inside the cave, keystone taxa consisted of archaeal groups of *Candidatus* Methanoperedens (*Euryarchaeota*), *Nitrosopumilaceae* (*Thaumarchaeota*) and bacterial groups of subgroup 6 (*Acidobacteria*), *Rokubacteriales* (*Rokubacteria*) and MB-A2-108 (*Actinobacteria*) (Figure 4D; Supplementary Table 5). Archaeal *Candidatus* Methanoperedens, mainly observed in SP samples, could anaerobically oxidize methane with nitrate as the electron acceptor *via* the reverse methanogenesis pathway and was enriched from an Italian paddy field (Vaksmaa et al., 2017). The ecological trait assignment analysis in SP further confirmed methanogenesis in SP samples with the water-flooded anaerobic conditions due to the convergence of water system at the entrance of the Heshang Cave (Figure 5A). Chemolithoautotrophic *Nitrosopumilaceae*, predominant in sediment, is able to oxidize ammonia in cave ecosystems (Hathaway et al., 2021). Acidobaterial subgroup 6 can be only enriched in the presence of *Alphaproteobacteria* and has been observed in a chemolithoautotrophically based cave ecosystem (Spring et al., 2000; Meisinger et al., 2007), suggesting that mutually beneficial trophic relationships among subgroup 6 and other taxa in caves. *Rokubacteriales*, dominated in sediment samples especially in the SD samples (11.93 ± 3.85%), may be capable of nitrogen respiration as indicated by their genomic information (Becraft et al., 2017), which is coincident with the results of ecological trait assignment dominated by nitrification and aerobic ammonia oxidation (Figure 5A).

Group 1.1c, *Nitrosophaeraceae*, and *Euryarchaeota* were the keystone taxa in the archaeal network outside the cave. Group 1.1c, dominant in acid FS soils (pH of 4.05 to 5.69), prefer acidic condition and have metabolic potential of fatty acid oxidation in deep anoxic peat (Lin et al., 2015). *Nitrosophaeraceae*, enriched in FS (15.74 ± 9.89%), is known as nitrifiers in aerobic processes (Longepierre et al., 2021), which might play roles in nitrification and aerobic ammonia oxidation in FS niches (Figure 5A). Microbial functional inference further confirmed functional niche differentiation in cave ecosystems except ecological trait assignments (Figures 5B,C). Microbial functional inferences inside the cave was dominated by energy metabolism, such as adenylate cyclase and 8-oxo-dGTP diphosphates, and ATP binding functions, which were closely correlated to DW niches (Figure 5C). In contrast, microbial functional inferences in overlying soils were related to carbohydrate metabolism such as dehydrogenase of aldehyde, long-chain acyl-CoA synthetase, succinate-semialdehyde/glutarate-semialdehyde, and 3-hydroxybutyrate, biotin metabolism and valine, leucine and isoleucine degradation (Figure 5C). It has been reported that most microbes exhibit energy metabolism due to oligotrophic conditions inside caves (Ortiz et al., 2014; Wiseschart et al., 2019), whereas carbohydrate metabolism, biotin metabolism, and degradation were found to be the pivotal functional inferences in outside soil ecosystems in our study.

## 5. Conclusion

Our results demonstrated differences in community structure, correlation with environmental factors, keystone taxa and potential ecological functions, and interactions between archaea and bacteria, providing new knowledge on archaeal ecology in subterranean biosphere.

1. Both archaeal and bacterial communities showed significant differences in their composition and correlations with environmental factors inside the cave and outside the cave. *Thermoplasmata*, *Nitrosopumilaceae*, *Aenigmarchaeales*, *Crossiella*, *Acidothermus*, and *Solirubrobacter* inside the cave were significantly distinguished from those in overlying soils outside the cave and these members were closely correlated to NH_4_^+^ concentration. In contrast, *Candidatus* Nitrososphaera, *Candidatus* Nitrocosmicus, *Thaumarchaeota* Group 1.1c, MB-A2-108, and *Pseudonocardiaceae* served as the predictors for microbial communities in the overlying karst soil ecosystems, which correlated to pH, Ca^2+^, and NO_2_^−^ concentrations.
2. Archaea were more connected than bacteria as indicated by the network analysis. The topological properties of bacterial networks were similar to those of the total prokaryotic networks.
3. Chemolithoautotrophic keystone taxa in the subsurface networks such as subgroup 6, *Candidatus* Methanoperedens, *Rokubacteriales*, and *Nitrosopumilaceae,* might maintain the stability of the cave ecosystem *via* obtaining energy under oligotrophic conditions. On the contrary, microbial functions in karst soils mainly involved in carbohydrate metabolism, biotin metabolism, and synthesis of fatty acid. Notably, all the keystone taxa were correlated to nitrogen cycle in karst ecosystems, indicating the fundamental role of nitrogen in the subsurface biosphere.

## Data availability statement

The datasets presented in this study can be found in online repositories. The names of the repository/repositories and accession nusmber(s) can be found at: https://www.biosino.org/node, OER254939.

## Author contributions

XC analyzed the data and wrote the manuscript draft. XX assisted in data analysis. YY completed the sample collection and DNA extraction. WW assisted with the partial data analysis. HW organized and conducted the sampling campaigns, obtained funded projects and supervised manuscript. PB assisted in reviewing the manuscript. All authors contributed to the article and approved the submitted version.

## Funding

This work was jointly supported by the National Natural Science Foundation of China (grant numbers 91951208 and 41130207) and China Scholarship Council (grant no. 202106410099).

## Conflict of interest

The authors declare that the research was conducted in the absence of any commercial or financial relationships that could be construed as a potential conflict of interest.

## Publisher’s note

All claims expressed in this article are solely those of the authors and do not necessarily represent those of their affiliated organizations, or those of the publisher, the editors and the reviewers. Any product that may be evaluated in this article, or claim that may be made by its manufacturer, is not guaranteed or endorsed by the publisher.

## Footnotes

^1^ https://www.biosino.org/node

^2^ https://cran.r-project.org/web/packages/vegan/index.html

^3^ https://cran.r-project.org/web/packages/randomForest/index.html

^4^ https://cran.r-project.org/web/packages/A3/index.html

^5^ http://www.loucalab.com/archive/FAPROTAX/lib/php/index.php?section=Download

^6^ https://cran.r-project.org/web/packages/pheatmap/index.htm

^8^ https://cran.r-project.org/web/packages/ggplot2/index.html

^9^ https://cran.r-project.org/web/packages/Hmisc/index.html

^10^ https://cran.r-project.org/web/packages/igraph/index.html
