# Supplementary material for: Archaea and their interactions with bacteria in a karst ecosystem
Source: Front Microbiol. 2023 Feb 6;14:1068595. doi: 10.3389/fmicb.2023.1068595 (PMC9939782; doi:10.3389/fmicb.2023.1068595)
Supplement: Supplementary file 1 [file Data_Sheet_1.docx]

Supplementary Material

## Supplementary figures


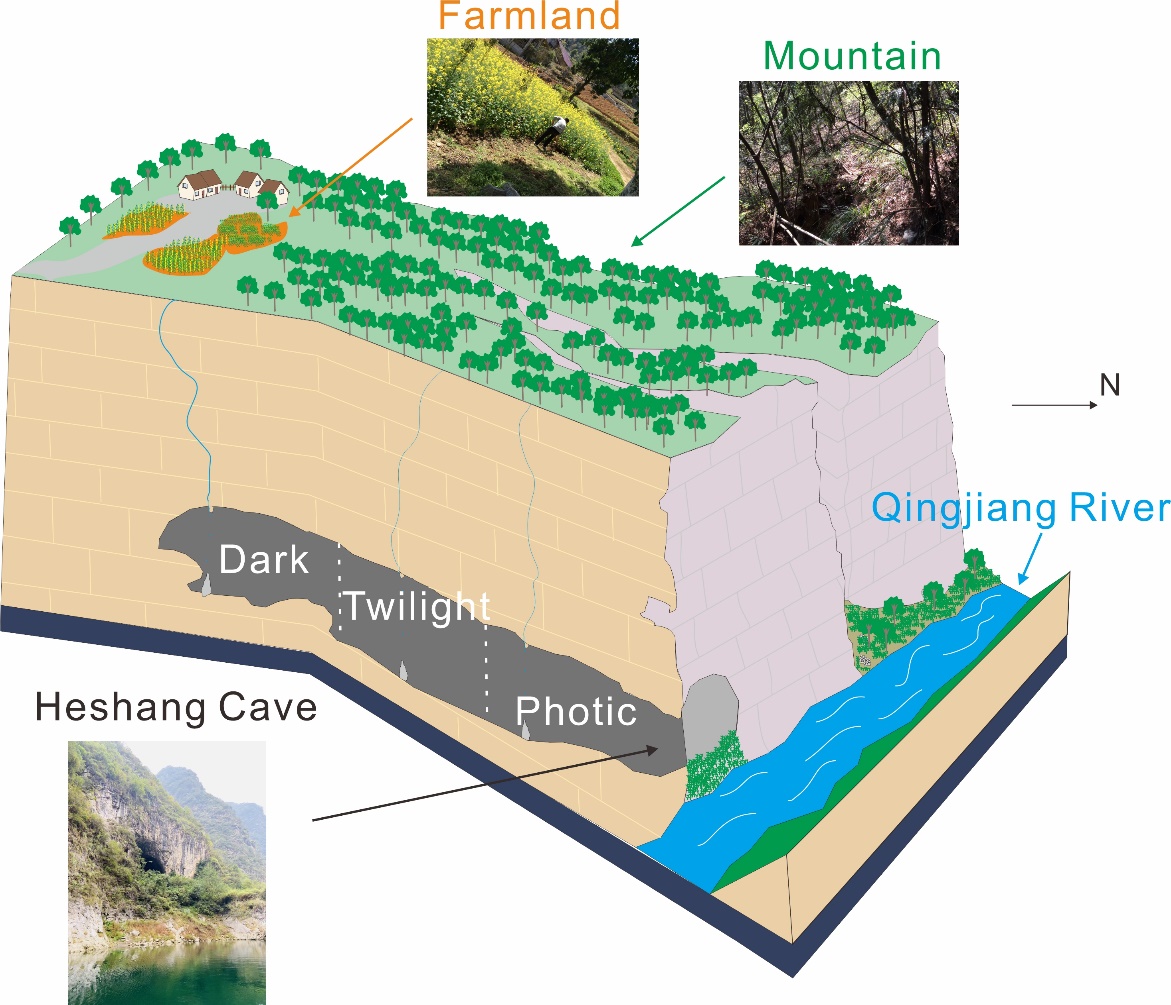


**Supplementary Figure 1. Schematic diagram of Heshang Cave karstic system, Hubei Province, central China.**


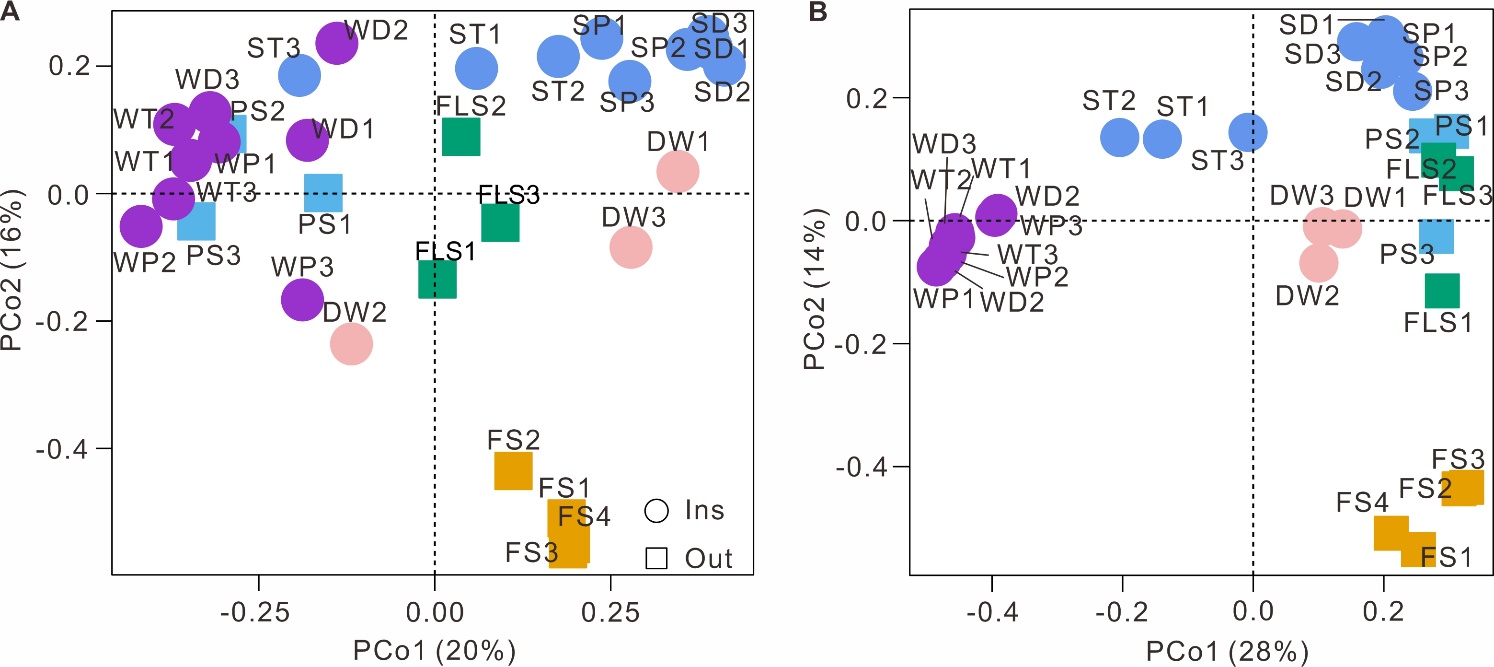


**Supplementary Figure 2.** Principal coordinate analysis (PCoA) plots of the archaeal (A) and bacterial (B) community structures in Heshang Cave karstic system, Hubei Province, central China. Ins, inside cave; Out, outside cave. FS, forest soil; PS, pristine soil; FLS, farmland soil; WD, weathered rocks in dark zone; WT, weathered rocks in twilight zone; WP, weathered rocks in photic zone; SD, sediments in dark zone; ST, sediments in twilight zone; SP, sediments in photic zone; DW, drip waters.

## Supplementary tables

**Supplementary Table 1 MRPP, ANOSIM, and Adonis pairwise comparison of archaeal and bacterial communities based on Bray-Curtis distance matrix (permutations = 9999) in Heshang Cave ecosystem, central China.**

|  |  | MRPP | |  | ANOSIM | |  | Adonis | |
| --- | --- | --- | --- | --- | --- | --- | --- | --- | --- |
|  |  | δ | *p* |  | *r* | *p* |  | *F* | *p* |
| Archaea | In × Out | 0.79 | 0.002 |  | 0.41 | 0.002 |  | 0.10 | 0.001 |
| Bacteria | In × Out | 0.74 | 0.001 |  | 0.46 | 0.001 |  | 0.18 | 0.001 |

**Supplementary Table 2 The relative abundance (%) of archaeal and bacterial phylum in Heshang Cave ecosystem.**

|  | Outside | | |  | Inside | | | | | | | Outside  (*p*) | Inside  (*p*) |
| --- | --- | --- | --- | --- | --- | --- | --- | --- | --- | --- | --- | --- | --- |
| Archaea_phylum | PS | FLS | FS |  | DW | SD | ST | SP | WD | WT | WP |  |  |
| ***Thaumarchaeota***** | 3.93 ± 1.50 | 18.21 ± 11.82 | 0.23 ± 0.14 |  | 5.48 ± 3.30 | 21.69 ± 8.88 | 7.96 ± 3.72 | 10.98 ± 3.10 | 5.43 ± 1.45 | 3.07 ± 1.05 | 3.15 ± 2.33 | ***** | ****** |
| ***Euryarchaeota**** | 0.04 ± 0.04 | 0.29 ± 0.20 | 0.06 ± 0.04 |  | 9.15 ± 12.40 | 12.08 ± 2.35 | 2.28 ± 2.38 | 2.16 ± 0.81 | 0.19 ± 0.16 | 0.19 ± 0.19 | 0.17 ± 0.23 | ns | ns |
| ***Nanoarchaeaeota***** | 0.005 ± 0.001 | 0.01 ± 0.006 | 0.001 ± 0.001 |  | 1.78 ± 1.33 | 0.17 ± 0.09 | 0.02 ± 0.009 | 0.51 ± 0.19 | 0.02 ± 0.01 | 0.009 ± 0.008 | 0.03 ± 0.03 | ns | ***** |
| *Crenarchaeota* | 0.009 ± 0.006 | 0.03 ± 0.006 | 0.02 ± 0.01 |  | 0.47 ± 0.60 | 0.88 ± 0.89 | 0.02 ± 0.0008 | 0.24 ± 0.10 | 0.04 ± 0.03 | 0.02 ± 0.0008 | 0.06 ± 0.07 | ns | ns |
| Others | 0.05 ± 0.03 | 0.06 ± 0.06 | 0.003 ± 0.002 |  | 0.12 ± 0.15 | 0.12 ± 0.04 | 0.08 ± 0.02 | 0.05 ± 0.03 | 0.03 ± 0.03 | 0.01 ± 0.009 | 0.01 ± 0.01 | ns | ns |
| *Asgardaeota* | / | 0.002 ± 0.002 | 0.005 ± 0.005 |  | 0.005 ± 0.005 | / | / | 0.0006 ± 0.0008 | 0.003 ± 0.005 | 0.0006 ± 0.0008 | 0.006 ± 0.009 | ns | ns |
| *Diapherotrites* | / | / | / |  | 0.004 ± 0.006 | / | / | / | / | / | / | ns | ns |
| *Hadesarchaeaeota* | / | / | / |  | / | / | 0.001 ± 0.002 | 0.001 ± 0.002 | / | / | 0.0006 ± 0.0008 | ns | ns |
| *Hydrothermarchaeota* | / | / | / |  | / | / | / | / | 0.002 ± 0.002 | / | 0.0006 ± 0.0008 | ns | ns |
| Bacteria_phylum |  |  |  |  |  |  |  |  |  |  |  |  |  |
| *Actinobacteria* | 76.01 ± 5.46 | 64.11 ± 15.49 | 81.98 ± 6.74 |  | 52.33 ±23.08 | 17.79 ± 5.39 | 71.77 ± 5.75 | 50.92 ± 6.02 | 88.92 ± 4.13 | 90.18 ± 2.31 | 88.72 ± 4.44 | ns | ****** |
| ***Rokubacteria***** | 8.09 ± 3.67 | 5.40 ± 2.55 | 6.87 ± 6.23 |  | 1.43 ± 1.81 | 24.22 ± 5.99 | 1.63 ±1.01 | 6.05 ± 2.22 | 0.46 ± 0.43 | 0.41 ± 0.15 | 0.25 ± 0.13 | ns | ****** |
| ***Firmicutes***** | 1.71 ± 0.47 | 3.07 ± 1.12 | 1.61 ± 0.71 |  | 4.21 ± 3.67 | 4.41 ± 2.92 | 11.71 ± 1.09 | 12.89 ± 7.18 | 2.99 ± 2.51 | 4.29 ± 2.02 | 2.67 ± 3.07 | ns | ns |
| ***Acidobacteria**** | 3.58 ± 2.70 | 5.26 ± 4.67 | 4.35 ± 2.21 |  | 0.47 ± 0.21 | 1.22 ± 0.33 | 0.49 ± 0.14 | 7.14 ± 3.44 | 0.14 ± 0.03 | 0.19 ± 0.04 | 0.23 ± 0.13 | ns | ****** |
| **GAL15**** | 0.03 ± 0.03 | 0.11 ± 0.08 | 0.04 ± 0.06 |  | 0.29 ± 0.29 | 13.03 ± 4.29 | 0.13 ± 0.03 | 1.26 ± 0.86 | 0.008 ± 0.004 | 0.009 ± 0.002 | 0.01 ± 0.005 | ns | ****** |
| *Cyanobacteria* | 1.56 ± 1.28 | 0.54 ± 0.18 | 1.35 ± 0.97 |  | 5.34 ± 6.15 | 0.06 ± 0.02 | 0.07 ± 0.06 | 1.65 ± 0.10 | 0.13 ± 0.15 | 0.05 ± 0.03 | 3.27 ± 2.87 | ns | ns |
| ***Proteobacteria***** | 0.04 ± 0.005 | 0.11 ± 0.07 | 0.28 ± 0.17 |  | 11.63 ± 2.83 | 0.04 ± 0.03 | 0.08 ± 0.07 | 0.38 ± 0.28 | 0.01 ± 0.005 | 0.05 ± 0.03 | 0.06 ± 0.03 | ns | ****** |
| ***Chloroflexi**** | 0.05 ± 0.03 | 0.27 ± 0.10 | 2.02 ± 1.65 |  | 0.01 ± 0.009 | 0.08 ± 0.03 | 0.12 ± 0.09 | 0.40 ± 0.16 | 0.01 ± 0.001 | 0.04 ± 0.02 | 0.10 ± 0.06 | ns | ****** |
| **BRC1**** | 0.40 ± 0.21 | 0.10 ± 0.09 | 0.004 ± 0.004 |  | 0.001 ± 0.0008 | 0.42 ± 0.18 | 0.14 ± 0.11 | 0.67 ± 0.20 | 0.02 ± 0.02 | 0.01 ± 0.004 | 0.008 ± 0.003 | ***** | ****** |
| ***Planctomycetes***** | 0.20 ± 0.07 | 0.07 ± 0.05 | 0.23 ± 0.11 |  | 0.02 ± 0.01 | 0.02 ± 0.01 | 0.02 ± 0.01 | 0.14 ± 0.09 | 0.002 ± 0.002 | 0.01 ± 0.01 | 0.02 ± 0.005 | ns | ***** |
| ***Omnitrophicaeota***** | 0.006 ± 0.008 | 0.002 ± 0.001 | 0.0008 ± 0.001 |  | 0.61 ± 0.45 | 0.02 ± 0.01 | 0.003 ± 0.002 | 0.06 ± 0.006 | / | 0.001 ± 0.002 | 0.0006 ± 0.0008 | ns | ***** |
| ***Patescibacteria***** | 0.01 ± 0.01 | 0.03 ± 0.02 | 0.08 ± 0.04 |  | 0.22 ± 0.12 | 0.006 ± 0.002 | 0.01 ± 0.005 | 0.11 ± 0.09 | 0.001 ± 0.001 | 0.01 ± 0.01 | 0.03 ± 0.01 | ns | ***** |
| **Others**** | 4.28 ± 1.08 | 2.32 ± 1.10 | 0.87 ± 0.28 |  | 6.43 ± 1.19 | 3.75 ± 1.17 | 3.44 ± 0.94 | 4.38 ± 1.24 | 1.59 ± 1.40 | 1.44 ± 0.62 | 1.19 ± 0.83 | ***** | ****** |

Statistically significant results are in bold face: *, 0.01 < *p* < 0.05; **, *p* < 0.01. FS, forest soil; PS, pristine soil; FLS, farmland soil; WD, weathered rocks in dark zone; WT, weathered rocks in twilight zone; WP, weathered rocks in photic zone; SD, sediments in dark zone; ST, sediments in twilight zone; SP, sediments in photic zone; DW, drip waters.

**Supplementary Table 3 The correlation between environmental parameters and microbial community with Mantel test under the permutations of 9999 inside and outside Heshang Cave.**

|  | Archaea_inside | Bacteria_inside |  | Archaea_outside | Bacteria_outside |
| --- | --- | --- | --- | --- | --- |
| Environmental factors | -0.04 | -0.14 |  | 0.12 | **0.34*** |
| pH | 0.02 | -0.05 |  | **0.72**** | **0.77**** |
| TOC | 0.15 | 0.13 |  | -0.03 | 0.18 |
| Ca^2+^ | -0.14 | -0.18 |  | 0.17 | **0.37*** |
| Mg^2+^ | -0.11 | -0.19 |  | -0.15 | 0.02 |
| K^+^ | 0.09 | -0.06 |  | 0.11 | 0.10 |
| Na^+^ | -0.11 | -0.19 |  | -0.07 | -0.08 |
| NH_4_^+^ | 0.04 | -0.07 |  | -0.11 | 0.17 |
| Cl^-^ | -0.06 | -0.11 |  | -0.07 | -0.18 |
| NO_2_^-^ | 0.03 | -0.12 |  | 0.18 | **0.41*** |
| NO_3_^-^ | -0.15 | -0.16 |  | 0.05 | 0.15 |
| SO_4_^2-^ | -0.02 | -0.12 |  | -0.09 | 0.06 |

**Supplementary Table 4 Topological properties of microbial occurrence networks in Heshang Cave ecosystem, central China.**

|  | Nodes | Links | Positive links (%) | Negative links (%) | APL | ACC | Diam | Modularity | Density | AD | AWD |
| --- | --- | --- | --- | --- | --- | --- | --- | --- | --- | --- | --- |
| Archaea_inside | 250 | 1405 | 97.79 | 2.21 | 3.95 | 0.45 | 12 | 0.44 | 0.05 | 11.24 | 8.76 |
| Bacteria_inside | 181 | 521 | 93.75 | 6.25 | 4.96 | 0.40 | 16 | 0.57 | 0.03 | 5.76 | 4.88 |
| Inside archaea and bacteria | 194 | 523 | 90.79 | 9.21 | 4.96 | 0.40 | 17 | 0.60 | 0.03 | 5.39 | 4.58 |
| Archaea_outside | 36 | 80 | 98.44 | 1.56 | 3.40 | 0.61 | 9 | 0.56 | 0.13 | 4.44 | 3.98 |
| Bacteria_outside | 145 | 128 | 90.25 | 9.75 | 1.58 | 0.54 | 4 | 0.86 | 0.01 | 1.77 | 1.75 |
| Outside archaea and bacteria | 152 | 132 | 98.48 | 1.52 | 1.57 | 0.53 | 4 | 0.87 | 0.01 | 1.74 | 1.72 |

APL, average path length; ACC, average clustering coefficient; Diam, diameter; AD, average degree; AWD, average weighted degree.

**Supplementary Table 5 The taxonomy of top 5 keystone taxa in the co-occurrence networks of Heshang Cave, central China**

|  | OTU number | BC | Taxonomy |
| --- | --- | --- | --- |
| Archaea_inside | OTU24508 | 3337.34 | p_*Euryarchaeota*; c_*Methanomicrobia*; o_*Methanosarcinales*; f_*Methanoperedenaceae*; g_*Candidatus* Methanoperedens |
|  | OTU40480 | 2070.90 | p_*Thaumarchaeota*; c_*Nitrososphaeria*; o_*Nitrosopumilales*; f_*Nitrosopumilaceae* |
|  | OTU600 | 1953.95 | p_*Thaumarchaeota*; c_*Nitrososphaeria*; o_*Nitrosopumilales*; f_*Nitrosopumilaceae* |
|  | OTU38158 | 1916.74 | p_*Euryarchaeota*; c_*Thermoplasmata* |
|  | OTU15659 | 1743.24 | p_*Euryarchaeota*; c_*Methanobacteria*; o_*Methanobacteriales*; f_*Methanobacteriaceae*; g_*Methanobacterium* |
| Bacteria_inside | OTU42103 | 3304.92 | p_*Acidobacteria*; c_Subgroup6 |
|  | OTU17863 | 2865.37 | p_*Rokubacteria*; c_*Rokubacteriales* |
|  | OTU30604 | 2310.73 | p_*Actinobacteria*; c_MB-A2-108 |
|  | OTU20080 | 1567.32 | p_*Acidobacteria*; c_Subgroup9 |
|  | OTU23090 | 1526.19 | p_*Actinobacteria*; c_*Thermoleophilia*; o_*Gaiellales* |
| Inside | OTU42103 | 3996.59 | p_*Acidobacteria*; c_Subgroup6 |
|  | OTU17863 | 3111.41 | p_*Rokubacteria*; c_*Rokubacteriales* |
|  | OTU30604 | 2233.34 | p_*Actinobacteria*; c_MB-A2-108 |
|  | OTU23517 | 1792.17 | p_*Rokubacteria;* c_*Rokubacteriales* |
|  | OTU23090 | 1616.19 | p_*Actinobacteria*; c_*Thermoleophilia*; o_*Gaiellales* |
| Archaea_outside | OTU18674 | 212.75 | p_*Thaumarchaeota*; c Group_1.1c |
|  | OTU36696 | 205.13 | p_*Thaumarchaeota*; c_*Nitrososphaeria*; o_*Nitrososphaerales*; f_*Nitrososphaeraceae* |
|  | OTU12085 | 150.00 | / |
|  | OTU42617 | 112.35 | p_*Thaumarchaeota*; c_*Nitrososphaeria*; o_*Nitrososphaerales*; f_*Nitrososphaeraceae* |
|  | OTU13161 | 87.17 | / |
| Bacteria_outside | OTU9369 | 12.00 | p_*Actinobacteria*; c_*Thermoleophilia*; o_*Solirubrobacterales*; f_67-14 |
|  | OTU25441 | 11.23 | / |
|  | OTU32492 | 11.23 | p_*Actinobacteria*; c_*Thermoleophilia*; o_*Solirubrobacterales*; f_67-14 |
|  | OTU4592 | 11.00 | p_*Actinobacteria*; c_*Acidimicrobiia*; o_*Microtrichales*; f_*Ilumatobacteraceae*; g_*Ilumatobacter* |
|  | OTU38484 | 9.00 | p_*Actinobacteria*; c_*Thermoleophilia*; o_*Gaiellales* |
| Outside | OTU9369 | 12.00 | p_*Actinobacteria*; c_*Thermoleophilia*; o_*Solirubrobacterales*; f_67-14 |
|  | OTU25441 | 11.23 | / |
|  | OTU32492 | 11.23 | p_*Actinobacteria*; c_*Thermoleophilia*; o_*Solirubrobacterales*; f_67-14 |
|  | OTU4592 | 11.00 | p_*Actinobacteria*; c_*Acidimicrobiia*; o_*Microtrichales*; f_*Ilumatobacteraceae*; g_*Ilumatobacter* |
|  | OTU38484 | 9.00 | p_*Actinobacteria*; c_*Thermoleophilia*; o_*Gaiellales* |

BC, betweenness centrality.
